# Uniform quantification of single-nucleus ATAC-seq data with Paired-Insertion Counting (PIC) and a model-based insertion rate estimator

**DOI:** 10.1038/s41592-023-02103-7

**Published:** 2023-12-04

**Authors:** Zhen Miao, Junhyong Kim

**Affiliations:** 1 Graduate Group in Genomics and Computational Biology, Perelman School of Medicine, University of Pennsylvania, Philadelphia, PA USA; 2 Department of Biology, University of Pennsylvania, Philadelphia, PA USA

**Keywords:** Software, Statistical methods, Epigenomics, Computational models

## Abstract

Existing approaches to scoring single-nucleus assay for transposase-accessible chromatin with sequencing (snATAC-seq) feature matrices from sequencing reads are inconsistent, affecting downstream analyses and displaying artifacts. We show that, even with sparse single-cell data, quantitative counts are informative for estimating the regulatory state of a cell, which calls for a consistent treatment. We propose Paired-Insertion Counting as a uniform method for snATAC-seq feature characterization and provide a probability model for inferring latent insertion dynamics from snATAC-seq count matrices.

## Abstract (web summary)

This study demonstrates the need and advantage of uniformly quantifying single-nucleus ATAC-seq data using Paired-Insertion Counting.

## Main

**Fig. 1:**
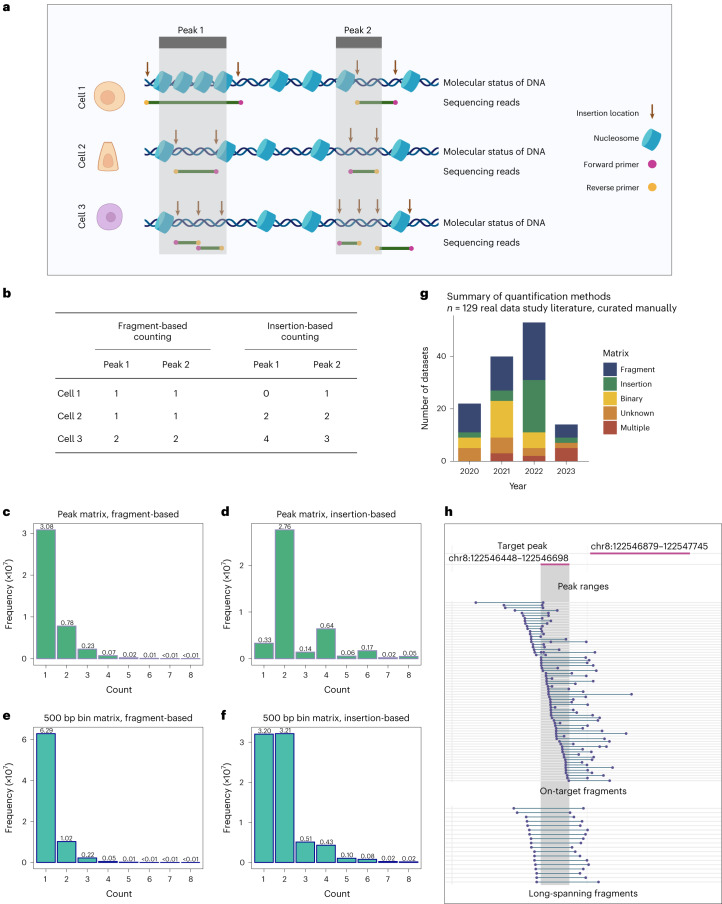
Existing counting strategies for snATAC-seq data processing are inconsistent. **a**, Schematic example of relationships between Tn5 insertion configuration and sequencing reads vis-à-vis peak location. **b**, Readout of insertion-based or fragment-based counting strategies for the example in **a**. **c**–**f**, Histograms of count frequencies with two counting strategies (fragment-based (**c**,**e**) and insertion-based (**d**,**f**)) and with peaks (**c**,**d**) or 500-bp bins (**e**,**f**) as features (PBMC-5k data^11^). The values indicated above the bars represent count frequencies (in 10^7^ units) in the data matrix. **g**, Number of datasets analyzed with binary, fragment or insertion matrices among 129 publications from 2020 to June 2023. The input matrix is curated from the methods section of the literature (Supplementary Table 2). **h**, Example peak with inconsistent DAR results between two counting strategies, from the P0 mouse kidney snATAC-seq dataset^17^. The target peak and an adjacent peak are shown. Fragments were classified into on-target fragments, where both counting strategies output nonzero count, and long-spanning fragments, where insertion-based counting outputs zero count but fragment-based counting outputs nonzero counts. Additional examples are shown in Supplementary Fig. 1c,d.

snATAC-seq assays open chromatin profiles of individual cells by amplifying genomic fragments between pairs of transposon inserts. The first step of ATAC-seq analysis, after choosing bins or peaks as region of interest (ROI), is to assign the feature counts based on either the number of fragments that overlap with an ROI (fragment-based counting; for example, Signac^1^ and snapATAC^2^) or the number of insertions (insertion-based counting; for example, 10× Cell Ranger ATAC^3^ and ArchR^4^). After feature counting, most methods convert the counts into a binary state of ‘open’ or ‘closed’^2,5–8^, while others retain quantitative count information, implying that single-nucleus assays might contain quantitative information on nucleosome density^4,9,10^. Unfortunately, as shown below, these different approaches to counting snATAC-seq peaks/bins lead to inconsistent quantification and downstream results (Fig. 1a,b), which is evident from the histograms of counts for fragment-based or insertion-based counting applied to the same dataset^11^ (Fig. 1c–f and Supplementary Table 1).

In a standard ATAC-seq experiment, two Tn5 insertions with an appropriate adapter configuration are required to form one amplicon fragment; thus the unit of observation is pairs of insertions. With insertion-based counting, there is an artifact of depleted odd numbers (Fig. 1c–f). Odd number of insertions arise only when fragments span across peak boundaries, artificially breaking up paired insertions of a fragment. Another issue of insertion-based counting occurs when two adjacent fragments share an insertion end. In the current insertion-based counting workflow, each read is processed independently, and the information of shared insertion is ignored and counted twice (Supplementary Note 1). Fragment-based counting also has problems of false positives when long fragments arise from two insertions that are spaced widely apart (for example, cell 1 in Fig. 1a). Such long fragments may indicate accessibility of two independent regulatory regions (on both ends), but it is unclear whether the region in between these loci is also accessible. This issue is particularly acute for specialized technologies like single-cell transposome hypersensitive sites sequencing (scTHS-seq)^12,13^ and scNanoATAC-seq^14^. Consequently, current fragment-based counting methods may lead to false positives counts when insertions are distantly outside the peak/bin^15,16^. The discrepancy is more pronounced when features are set to fixed-size bins, as the region boundaries are arbitrary.

The different counting strategies can result in discrepancies in downstream analysis. As an example, we analyzed a P0 mouse kidney snATAC-seq dataset^17^ for differentially accessible region (DAR) identification with ArchR^4^ and Signac^1^ (Methods). Using fragment- or insertion-based counting or binary input, we found that, for the same input data, up to 4.7% of the peaks in the DAR set are inconsistent (Supplementary Fig. 1a,b). Example peaks with inconsistent DAR results are shown where long-spanning fragments result in counts of zero with insertion counting versus one with fragment counting (Fig. 1h and Supplementary Fig. 1c,d).

Due to these discrepancies, data matrices processed with different quantification approaches cannot be combined directly, thereby impeding data integration. We compiled 129 recently published datasets and found that all three types—fragment count, insertion count and binary count (reduction to 0/1)—have been employed frequently for data processing, with certain studies employing more than one type (Fig. 1g and Supplementary Table 2). With increasing need for data integration and reproducibility in scientific investigations, establishing a uniform counting method is a critical need.

**Fig. 2:**
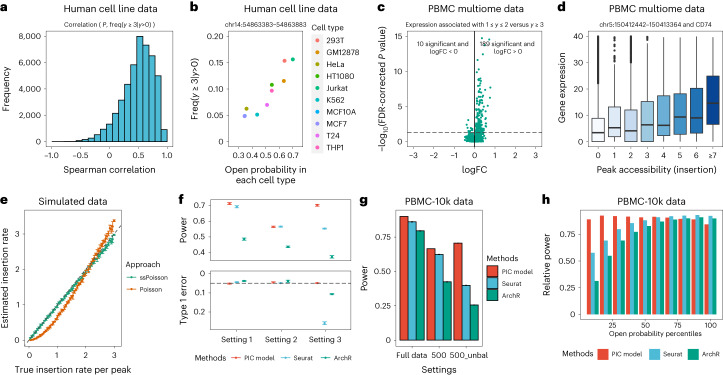
snATAC-seq data contain quantitative information of cellular states. **a**, Histogram of Spearman correlation coefficients between the probability of accessible peak in each group and the relative frequency of high-density insertion counts in human cell line data. **b**, Example peak with correlated open probabilities and relative frequency of peaks with high-density insertion across cell types in the human cell line data. **c**, Volcano plot showing the normalized gene expression levels between cells with TSS peak insertion counts equal to 1 or 2 and cells with high-density TSS peak insertion counts in PBMC data. Two-sided Wilcoxon rank sum test was used for the comparison and FDR correction was used to adjust for multiple comparisons. logFC, log fold change. The horizontal dashed line represents the (FDR corrected) *P* value of 0.05. **d**, An example of peak–gene pair where normalized gene expression levels are related to the TSS peak insertion counts in PBMC data; *n* = 10,538 cells were examined over one independent experiment. Center line in box plot represents median and the lower and upper hinges correspond to the first and third quartiles. The upper or lower whisker corresponds to 1.5 times the interquartile range or the largest/smallest values. **e**, Relationship between estimated insertion rate with simulation data and the true insertion rate, under size-filtered signed Poisson (ssPoisson) or standard Poisson distribution. Paired-Insertion counts from *n* = 500 cells were simulated; error bars represent the s.d. of the parameter estimation across five rounds of simulation. **f**, Power and type I error of PIC model, Seurat and ArchR DAR tests under three different simulation settings (see main text). Paired-Insertion counts from *n* = 500 cells in each group were simulated; error bars represent the s.d. of the parameter estimation across five rounds of simulation. The horizontal dashed line represents the nominal set significance value of 0.05. Methods that effectively control type 1 error should be above the line. **g**, Power of PIC model, Seurat and ArchR DAR tests under different settings with PBMC multiome data. 500_unbal represents the condition when 500 cells in each group are sampled, but with different mean capturing rate; error bars represent the s.d. of the parameter estimation across five rounds of simulation. **h**, Power of PIC model, Seurat and ArchR DAR tests for different rank percentiles of insertion rates for the full PBMC dataset.

If the counts are binarized, the insertion and fragment counting are mostly consistent with each other. So, we aimed to determine whether snATAC-seq data support quantitative information that would be lost with binary counts. We first asked whether more fragments in a peak for a single cell indicates higher probability that a randomly selected cell of the same type would be in the open state. We first analyzed a human cell line snATAC-seq dataset^4^ with insertion-based counting. For each peak, we calculated the relative proportion of cells with high-density peaks (no less than two fragments, that is, at least three insertions) for each of the ten cell types, and then compared their rank order with the rank order of the proportion of cells with accessible peaks (for each cell type) by Spearman rank correlation. For human cell line data, more than 94.6% peaks showed positive correlation and 9.4% showed significant correlation after false discovery rate (FDR) *P* value correction (34.5% without FDR correction; Fig. 2a; example peaks Fig. 2b and Supplementary Fig. 2a). Consistent results were found for other datasets (Supplementary Fig. 2b–d), including the sparser sci-ATAC-seq data.

Using multiome data^18^, we also examined the relationship between quantitative snATAC-seq count in promoter regions and the expression of corresponding genes in the same cell. We compared the gene expression levels of genes with a proximal transcript start site (TSS) peak insertion count = 1 or 2 (single fragment) against those with a count ≥ 3 (more than one fragment) using the Wilcoxon rank sum test. We found 199 significant peak–gene pairs after FDR correction, 189 of which have positive log fold change (Fig. 2c); 67.2% of peak–gene pairs showed higher nonzero expression proportion in the group with count ≥ 3. Figure 2d and Supplementary Fig. 2e show examples of peak–gene pairs where the distribution of RNA expression changes monotonically as a function of ATAC counts. Consistent results were found for other datasets (Supplementary Fig. 2f–h). In sum, we see quantitative information in the sparse snATAC-seq data. Such quantitative information in snATAC-seq data may arise from different level of accessibility of one regulatory element, or several subpeaks with near-binary accessibility, and our analyses suggest the former to be more common (Supplementary Note 2).

Since the direct evidence of open chromatin is at the insertion site, snATAC-seq quantification should be based on the insertion site but taking into account the problems noted above. Here, we propose a simple uniform counting strategy, Paired-Insertion Counting (PIC), that corrects for the peak boundary problem (Methods) along with a probability model that accounts for shared insertion points.

In PIC, for a given chromosome interval, if the pair of insertions of an ATAC-seq fragment are both within the interval, they are counted as one (pair); if only one insertion is within the interval and the other is outside the interval, also count one (pair).

PIC is consistent with the fact that all fragments have two insertions. It prevents counting a fragment when its ends are both outside the peak/bin interval. PIC is also valid for other single-cell open chromatin assays, including sci-ATAC-seq^19^, dscATAC-seq^20^, scTHS-seq^13^ and scNanoATAC-seq^14^.

To make optimal use of the quantitative information in snATAC-seq data, we propose a probability model that we call ‘PIC model’ to incorporate the molecular process of snATAC-seq fragment generation. We assume that a genomic interval has uniform per base pair insertion probability, resulting in Poisson counting events. But, given a Poisson distribution of insertions, the sequenced fragments are a subset of the insertions because: (1) inserted transposons must match in primer configurations^21^ and (2) there is a size selection on the fragments due to constraints in amplification, library construction and alignment. These two factors contribute to the higher sparsity of snATAC-seq data than those expected from the Poisson model.

Let $X$ be a random variable representing the number of insertions in a given peak and $W$ be a random variable representing PCR-amplifiable fragment. From the assumption of Poisson insertions, we have

1 $$\begin{array}{l}P\left(W=m\right)=\mathop{\sum }\limits_{n=0}^{{{\infty }}}P\left(W=m|X=n\right)P\left(X=n\right)\\ \,\qquad\qquad\quad=\left\{\begin{array}{c}\mathop{\sum }\limits_{n=m+1}^{{{\infty }}}\left(\begin{array}{c}n-1\\ m\end{array}\right){\left(\frac{1}{2}\right)}^{n-1}\frac{{\lambda }^{n}{\mathrm{e}}^{-\lambda }}{n!},\, \, \,\text{if}\,m > 0\\ {{\mathrm{e}}}^{-\lambda }+\mathop{\sum }\limits_{n=1}^{{{\infty }}}{\left(\frac{1}{2}\right)}^{n-1}\frac{{\lambda }^{n}{{\mathrm{e}}}^{-\lambda }}{n!},\qquad\, \, \, \, \,\text{if}\,m=0\end{array}\right.\end{array}$$

where $\lambda$ is the rate parameter in the Poisson distribution representing the average rate of insertions within a peak region. Adding the constraint of experimental size selection, the probability of observing *k* fragments is:

2 $$\begin{array}{l}P\left({W}_{S}=k\right)\\={\sum }_{m=k}^{{{\infty }}}\left(\begin{array}{c}m\\ k\end{array}\right){\left(\frac{{{\mathrm{e}}}^{-\frac{{s}_{1}\lambda }{{L}_{p}}}-{{\mathrm{e}}}^{-\frac{{s}_{2}\lambda }{{L}_{p}}}}{1-{{\mathrm{e}}}^{-\lambda }}\right)}^{k}{\left(1-\frac{{{\mathrm{e}}}^{-\frac{{s}_{1}\lambda }{{L}_{p}}}+{{\mathrm{e}}}^{-\frac{{s}_{2}\lambda }{{L}_{p}}}}{1-{{\mathrm{e}}}^{-\lambda }}\right)}^{m-k}P(W=m)\end{array}$$

where *W*_*S*_ is the random variable for size-selected fragments, *s*_1_ is the minimum size of fragment, *s*_2_ is the maximum size of fragment and *L*_*p*_ is the length of the peak. The hyperparameters *s*_1_ and *s*_2_ are estimated empirically from data as described in Methods. We call the distribution specified by equation (2) ‘size-filtered signed Poisson (ssPoisson)’ distribution. Our theoretical ssPoisson distribution well approximates distributions of simulated ATAC-seq experiments (Supplementary Table 3). Using simulated data, we found that our model provides a better estimate the true insertion rates than the Poisson model (Fig. 2e).

With estimated parameters, we developed a generalized likelihood ratio test based on PIC model for detecting DARs between different groups of cells (Methods). To evaluate the performance of this method, we conducted data simulation to test the type I error and power across a wide range of true insertion rates and capturing rates. Here three settings were evaluated: (1) two groups with equal number of cells and equal number of up- or downregulated peaks, (2) two groups with unequal number of cells and (3) two groups with equal number of cells but more upregulated peaks than downregulated peaks (Fig. 2f). Our method shows consistently high power while the type I error rates are under control; however, Seurat method has strong type I error inflation in setting (3) and ArchR has overall lower power. We next conducted comparison using the empirical peripheral blood mononuclear cells (PBMC) dataset^18^. We assessed type I error by label randomization and power by setting the consensus of all three methods as the (pseudo) true differential peak (Methods). The PIC model-based test showed an increase of 4.5% and 13.1% in the identification of DARs compared with Seurat and ArchR, respectively. In the setting of unbalanced subsamples, the distinction is more pronounced, with our test detecting 77.8% and 178.0% more DARs than Seurat and ArchR, respectively (Fig. 2g). PIC model is especially more powerful for peaks with overall low insertion rates, as expected from the theoretical distribution (Fig. 2h). Additional analysis of kidney P0 datasets^17^ and human brain data with SNARE-seq2 protocol^22^ obtained similar results (Supplementary Figs. 3–5). Assessment of binding motifs found within DAR peaks resulted in discovery of regulatory dynamics consistent with multiomics gene expression and the literature (Supplementary Note 3).

In addition to DAR, we explored the effect of quantification approaches on other downstream inferences (Supplementary Note 4) and found PIC framework improves inferences for ROI where the reads are quantitative.

Beyond our model, chromatin state and Tn5 insertion probability is likely to be governed by more complex molecular factors as shown in bulk ATAC-seq studies^15^ and aggregates of single cells^23^. Ideal inference of underlying chromatin accessibility states might benefit from a more comprehensive treatment of the biochemical factors and resulting transposon insertion patterns^23–25^. Nevertheless, there is a compelling need to summarize ATAC-seq data with a consistent procedure that allows broad downstream analyses. Addressing this, PIC provides a consistent approach, enabling consistent quantitative treatment of snATAC-seq data with a high-powered model-based DAR test.

We have made PIC modules publicly available (https://github.com/Zhen-Miao/PICsnATAC) and they can be incorporated easily into standard pipelines^1,4^.

## Methods

### Data quality control and preprocessing

For all datasets, we removed peaks with extensive instances of very high counts (≥7 with fragment-based counting or ≥14 with insertion-based counting) across the entire dataset, as these peaks could be associated with repetitive or potentially uncharacterized blacklist regions^2^. We removed potential doublet cells based on the number of regions with per base coverage greater than three^26^. We also removed fragments with interval length smaller than ten that are likely to be misalignment. The data sparsity, median sequencing depth and other metrics are reported in Supplementary Table 4.

### Processing 10x Genomics PBMC snATAC-seq data (5k)

The 10x Genomics PBMC snATAC-seq data (ID: atac_pbmc_5k_nextgem) were used to compare the count distribution obtained from different counting methods. The peak ranges and insertion-based peak-by-cell count matrices were obtained using the Cell Ranger ATAC pipeline (v.2.0.0) from 10x Genomics. Bins that are accessible in fewer than ten cells were filtered. The insertion-based bin-by-cell matrix was constructed by ArchR^4^ and the fragment-based matrix was constructed using Signac^1^.

### DAR detection with Seurat or ArchR

We used the P0 mouse kidney data to study the effects of different input on DAR analysis. The peak information as well as cell type annotations were obtained from the original publication^17^. The peak-by-cell matrix was then constructed with both insertion-based and fragment-based approaches. The count correspondence is summarized in Supplementary Table 5. We then focused on the two most abundant cell types—nephron progenitor cells and stroma cells—for the DAR analysis. Two approaches, Signac^1^ and ArchR^4^, were used to identify DARs. Specifically, Signac used logistic regression with a likelihood ratio test to identify DARs, a framework proposed by Ntranos et al.^27^. By using the group label as dependent variable, read count as independent variable and sequencing depth as a covariate, Signac identified peaks significantly predictive (different) of the two groups while adjusting for individual sequencing depth disparity. ArchR identified a subset of cells within each group so that the numbers of fragments in the two subsets were comparable, and then the Wilcoxon rank sum test was conducted on these subsets to compute DARs. By default, ArchR selects, at most, 500 cells from each group (‘maxCells = 500’), but here we set the value to 5,000 so all matched cells were selected (for fair comparison of power). For both methods, peaks with FDR-adjusted *P* value ≤0.05 were regarded as DARs.

### Zero-adjusted open probability estimation

We define ‘open probability’ as the probability that a given genomic region is accessible for a randomly sampled cell of a given cell type. Note that this open probability does not measure the degree of openness, but rather the probability of capturing a cell in an open state accessible to the ATAC-seq assay. This probability will be governed by the temporal dynamics of nucleosome-dependent accessibility of that region for that cell type. Typical snATAC-seq data have missing data issues and are very sparse. To unbiasedly estimate the chromatin open probability in each cell type, we considered two sources of zeros in the snATAC-seq data: biological inaccessibility and technical failure to capture open state in sequencing data. We developed the following model to estimate the true open proportion.

Let ${\mathbf{Z}}_{g}^{c}=({Z}_{g,\,1}^{c},\ldots ,{Z}_{g,\,J}^{c})^T$ be a *J* × 1 binary vector representing the open chromatin status of cell $c$ that depends on the group label $g$ (for example, cell type label). Each element in the vector ${Z}_{g,j}^{c}\in \{\mathrm{1,0}\}$ represents the accessibility of the *j*th genomic region (for example, bin or peak), where the value 1 indicates open and 0 indicates closed. We consider ${Z}_{g,j}^{c}$ to be sampled from a Bernoulli distribution parameterized by *p*_*g,j*_, the probability that a random cell of *g* type will be open for the *j*th region:

$${Z}_{g,\,j}^{c}\sim {\rm{Bernoulli}}\left({p}_{g,\,j}\right)$$

In practice, the true open chromatin status *Z* of cell $c$ is unobserved, since, due to disparity of enzyme activity and sequencing depth across cells, an open state may be masked due to missing data. We introduce ${{\mathbf{T}}}_{d}^{c}$ as a *J* × 1 binary vector representing the capture state of different genomic regions in cell $c$. This status depends on the sequencing depth *d* for cell *c*. Additional experimental factors and the particular chromosomal region may also affect the status, which we ignore here. We also drop index *d* since it is cell specific. We assume:

$${\mathbf{T}}^{c}\sim {\rm{Bernoulli}}({\mathbf{q}}^{c})$$

for some parameter vector **q**^*c*^ that is a function of the cell.

Let ${\mathbf{Y}}_{g}^{\,c}$ be a random vector representing the observed chromatin status of cell *c*. ${Y}_{g,j}^{c}\in \{\mathrm{0,1}\}$, where 1 indicates open and 0 indicates closed. Then ${\mathbf{Y}}_{g}^{c}={\mathbf{Z}}_{g}^{c}\otimes {\mathbf{T}}_{d}^{c}$ where $\otimes$ denotes the element-wise direct product (Hadamard product).

For a given dataset *y*, we set the loss function $\log {L}(\mathbf{p},\mathbf{q}|\mathbf{y})$ as

$$\log L(\mathbf{p},\mathbf{q}{\rm{|}}\mathbf{y})=\mathop{\sum }\limits_{j=1}^{J}\mathop{\sum }\limits_{c=1}^{C}[{\,y}_{{jc}}\log ({p}_{j}{q}_{c})+(1-{y}_{{jc}})\log (1-{p}_{j}{q}_{c})]$$

where the group label *g* is omitted. To compute both estimators for **p** and **q**, we implemented a coordinate descent algorithm. This iteration continues until convergence:

1. Start with an initial estimate of **p**^(0)^
2. For *t* = 1, 2,…
  - Compute $${q}_{c}^{(t)}$$ by:
    $${q}_{c}^{(t)}=\frac{{\sum }_{j=1}^{J}{y}_{{jc}}}{{\sum }_{j=1}^{J}{p}_{j}^{(t-1)}}$$
  - Update ${p}_{j}^{(t)}$ by moment estimator:

$${p}_{c}^{(t)}=\frac{{\sum }_{c=1}^{C}{y}_{{jc}}}{{\sum }_{c=1}^{C}{q}_{c}^{(t)}}$$

### Count frequency and open probability in human cell line data

The human cell line data matrix was constructed by the insertion-based counting method, and the maximum count was capped at 4 by the ArchR pipeline. We note that such a ceiling step does not affect our analysis. The open probability for each cell type, *p*_*g*_, was estimated with the method described above. Since the counts 2 and 1 represent mainly the boundary phasing issue, we estimated the probability of observing a count ≥3, given the observation of a nonzero count, *P*_*g*_[*y* ≥ 3│*y* > 0]

$${P}_{g}\left[\,y\ge 3\,|\,y > 0\right]=\frac{{f}_{3}+{f}_{4}}{{f}_{1}+{f}_{2}+{f}_{3}+{f}_{4}}\,$$

where *f*_*n*_ represents the frequency of count *n*.

Since some peaks do not have counts that are >3, we only retained peaks with ≥5 counts >3, and 46,499 peaks were left. The Spearman correlation was computed between the open probability and frequency of counts >3. In addition, we also computed the probability of observing a count = 2 given the count being 1 or 2, *P*_*g*_ [*y* = 2│*y* > 0]

$${P}_{g}\left[y=2\,|\,y=1\,{\mathrm{or}}\,2\right]=\frac{{f}_{2}}{{f}_{1}+{f}_{2}}\,$$

and its correlation with open probability.

### Count frequency and open probability in P0 mouse kidney data

We retained cell types with more than 600 cells to get accurate estimations of the parameters, which resulted in 9,286 cells across seven cell types. After quality control, we retained 256,574 peaks for the analysis. The count is not capped for this dataset. Within a cell type, the probability of observing counts ≥3, given the observation of a nonzero count, is estimated by

$${P}_{g}\left[\,y\ge 3\,|\,y > 0\right]=\frac{{f}_{3}+\cdots +{f}_{n}}{{f}_{1}+{f}_{2}+{f}_{3}+\cdots +{f}_{n}}$$

### Count frequency and open probability in human uterus sci-ATAC-seq data

Since the sci-ATAC-seq dataset is much smaller and sparser, we used a more lenient criteria when conducting filtering. We retained cell types with more than 50 cells, which resulted in eight cell types. We retained peaks with at least five counts >3, and 11,367 peaks were left. Analysis results for this dataset are shown in Supplementary Fig. 2c. We also tested a range of different filtering criteria, and consistent outcomes were observed.

### Count frequency and open probability in dscATAC-seq data

For the mouse brain dscATAC-seq data, two batches were included in the study (batch 1 and batch 2). In total, we obtained a data matrix of 454,047 peaks across 7,109 cells. We retained cell types with more than 200 cells, resulted in ten cell types. We retained peaks with at least ten counts >3, and 311,543 peaks were left. Analysis results for this dataset are shown in Supplementary Fig. 2d. We also tested a range of different filtering criteria, and consistent outcomes were observed.

### Gene expression and TSS insertion counts in PBMC multiome data

We first retained peaks that overlap with ±100 bp region around the TSS and with at least five instances of counts ≥2. Then, we linked these peaks with their associated genes to form peak–gene pairs. The peak–gene pairs were then filtered by requiring the nonzero expression proportion with chromatin insertion counts >0 to be at least 10%. A total of 3,387 such peak–gene pairs were kept for the downstream analysis.

For each peak–gene pair, we grouped the normalized gene expression levels by the insertion count in the TSS peak. Mean expression level and nonzero expression proportion were calculated for each group. Two-sided Wilcoxon rank sum test was then conducted between the two groups and log fold change was computed by comparing the mean expression differences.

### Gene expression and TSS insertion counts in BMMC data

The bone marrow mononuclear cells (BMMC) dataset^28^ was collected across several institutes and several donors with batch effect. To prevent batch effect, we focused on one donor sample that was collected at one institute (donor no. 2 collected from institute no. 1). There are 6,740 cells across several cell types. With the same filtration criteria as above, we retained 2,488 peak–gene pairs for our analysis. The same analyses as above were conducted and are shown in Supplementary Fig. 2f–h.

### PIC counting approach

To construct a cell-by-peak count matrix while preventing the issues with fragment-based or insertion-based counting, a PIC counting approach is proposed. PIC takes input from the fragment file (of which the first four columns should be chromosome, start, end and cell barcode), the filtered cell barcodes and a list of ROIs (that is, bins or peaks). Briefly, the two insertion loci for every fragment are obtained from the fragment file (‘start’ and ‘end’). Then, each insertion locus is mapped to the peak region. Such mapping can either assume that only the exact insertion location is accessible, the same as in insertion-based counting, or assume that a flanking window around insertion location is also accessible, as implied in MACS2-based peaks (usually specified as 37 bp or 75 bp). We recommend using the same flanking window size so that the quantification step is consistent with the peak calling step. If both insertion loci are mapped to the same ROI, we mask one of them and count only once.

With the 10x Genomics snATAC-seq assays, there are typically more than 20% reads (of PIC) that are greater than or equal to two, suggesting a substantial information loss with binarization. This proportion varies in different instruments/sequencing depths and is summarized in Supplementary Table 3. Within each single cell, the proportion of high-density peaks is a function of the in-peak fragment counts (Supplementary Fig. 6a–d).

For standard snATAC-seq data, PIC may have the drawback that, when one insertion is in the peak/bin and the other insertion is far away from the first insertion, the evidence is weak that both insertions provide information on the current peak/bin. However, in most datasets, long fragments with one insertion in the peak are rare and unlikely to greatly distort the data (Supplementary Fig. 7a–c). In general, when the peak intervals are large, all three methods will have nearly identical counts (after a twofold correction factor). However, when the peak intervals are small, say within the common range of fragment or bin lengths, or within the size range of estimated cis-regulatory elements, the three methods will diverge, and we propose that PIC will be most logically consistent and provide better quantitative information for downstream analyses.

For large datasets, PIC can also load the fragment files dynamically, enabled by Rsamtools^29^. Deduplication option is also provided, and is especially useful for dscATAC-seq data^3^.

### PIC model—size selection

In the ssPoisson model, the hyperparameters s1 and s2 can be estimated using data obtained from either the mapped reads themselves or Bioanalyzer traces. For example, in Supplementary Fig. 7, we presented the fragment length distribution for several datasets analyzed in the study. A key observation is the scarcity of fragments longer than 600 bp. Because ATAC fragments can encompass one or more nucleosomes, their length distribution is expected to exhibit local spikes at multiples of 200 bp (which is evident in the bulk assays). Therefore, the lack of a local spike in the read distribution around 600 bp suggests size selection, most likely attributed to the library preparation process. Thus, the default s2 value is set to 600 bp in our program. The hyperparameter s1, which stands for the lower limit of fragment length, is to ensure that the fragment should be amplifiable and mappable to the genome. The Cell Ranger ATAC report summary considers fragments longer than 25 bp (after the 9 bp correction of insertion overhang), and we set this as the default *s*_1_ value in the PIC framework. For specialized assays such as the scNanoATAC assay, long ATAC fragments are enriched for nanopore long-read sequencing; therefore *s*_2_ values should be adjusted. Overall, the distribution is more impacted by the *s*_1_ parameter than the *s*_2_ parameter (see the form of equation (2) in main text). For example, if *s*_1_ is set high, the distribution will diverge for larger expected number of insertions (because it will prevent close insertions). However, *s*_1_ should be set by the library protocol and alignments with the default value of 25 being a reasonable estimate. The effect of *s*_1_ and *s*_2_ on the expected read counts is shown in Supplementary Fig. 4d–e.

### PIC model—mean-variance relationship under condition 1

From the equation (1) (signed Poisson distribution), we can obtain the analytical expression for mean and variance

3 $$\mathrm{E}\left[W\right]=\frac{\lambda -1+{\mathrm{e}}^{-\lambda }}{2}$$

4 $${\mathrm{Var}}\left(W\right)=\frac{2\lambda +2{\mathrm{e}}^{-\lambda }-2\lambda {\mathrm{e}}^{-\lambda }-{\mathrm{e}}^{-2\lambda }-1}{4}$$

where the random variable *W* counts pairs of insertions. Equations (3) and (4) shows that the requirement for correct pair of primers results in a process with lower mean than the Poisson process, and the variance is larger than the mean (Supplementary Fig. 8a). We note that the variance can be larger or smaller than the mean in ssPoisson distribution (see Supplementary Table 3 for the simulation).

### PIC model—diploid cells

For diploid cells, we use *W*_*s*1_ and *W*_*s*2_ to denote the observed PIC count in two alleles, with insertion rates *X*_1_ and *X*_2_, respectively, and ${W}_{s}^{\prime} ={W}_{s1}+{W}_{s2}$ to denote the total observed PIC count in a cell. The Tn5 insertion events at either allele can be viewed as independent to one another, so we have:

5 $$P\left({W}_{s}^{\prime} =r\right)={\sum }_{k=0}^{r}\left[P\left({W}_{s1}=k\right)+P\left({W}_{s2}=r-k\right)\right]$$

Usually, we do not have the allele-specific fragment information but, under assumption of the same insertion rate for the two alleles, we can still estimate the rate parameter with moment estimator or maximum likelihood estimator, limiting equation (2) to finite terms.

### PIC model—insertion rate estimation

Assume we have a group of cells within one cell type (that is, they share the same underlying insertion rate for each peak), we can estimate the insertion rate with moment estimator or maximum likelihood estimator. Denote the observed PIC count after data missing as ${W}_{o}$, we have:

6 $$P\left({W}_{o}=t\right)=\mathop{\sum }\limits_{k=t}^{{{\infty }}}\left[ \left(\begin{array}{c}k\\ t\end{array}\right) {\left({q}_{i}\right)}^{t}{\left(1-{q}_{i}\right)}^{k-t}P\left({W}_{s}^{\prime} =k\right)\right]$$

7 $$\mathrm{E}\left[{W}_{o}\right]=\mathop{\sum }\limits_{t=1}^{{{\infty }}}t P\left({W}_{o}=t\right)={q}_{i} \mathop{\sum }\limits_{k=1}^{{{\infty }}}\left(k P\left({W}_{s}=k\right)\right)={q}_{i}\mathrm{E}[{W}_{s}]$$

where ${W}_{s}^{\prime}$ is the (theoretical) PIC count under condition 1 and 2, specified in equation (5), and *q*_*i*_ is the capturing rate of cell $i$ estimated using our estimation approach. Each term of the summation in equation (2) is a power law decreasing quantity; therefore, we approximate the expectation over a finite number of terms. Assume the total number of cells is $c$, their observed fragment counts are $\{{w}_{1},{w}_{2},\ldots ,{w}_{c}\}$, and the cell-specific capturing rates are $\{{q}_{1},{q}_{2},\ldots ,{q}_{c}\}$, missing-corrected mean PIC count is then ${\bar{\bar{w}}}=({\sum }_{i=1}^{c}{w}_{i}/{q}_{i})/c$. By taking the inverse of equation (7), we obtain the moment estimator of insertion rate, $\widehat{\lambda }$. To obtain the maximum likelihood estimator (MLE), ${\lambda }_{{\rm{MLE}}}$, we can use numerical optimization to obtain the maxima of the log likelihood function, ${\mathrm{LL}}(\lambda |{w}_{i},{q}_{i})$:

8 $${\mathrm{LL}}(\lambda {\rm{|}}{w}_{i},{q}_{i})=\mathop{\sum }\limits_{i=1}^{c}\log \left({P}_{\lambda }\left({W}_{o}={w}_{i}\right)\right)$$

### PIC model—statistical test for DARs

Assume we have two cell types indexed by {1,2,…,*c*_1_} and {*c*_1_ + 1, *c*_1_ + 2,…,*c*_1_ + *c*_2_}, here we propose a generalized likelihood ratio test for detecting peaks with different underlying insertion rate.

H_0_: the insertion rates for the two groups are identical.

H_1_: the insertion rates for the two groups are different.

We use M_0_: $\widehat{\lambda}_{c_1} = \widehat{\lambda}_{c_2} = \widehat{\lambda}$ to denote model under H_0_, and M_1_: ${\widehat{\lambda}}_{c_1} \neq {\widehat{\lambda }}_{c_2}$ to denote model under H_1_.

Log likelihoods:

9 $${\mathrm{LL}}_{{M}_{0}}=\mathop{\sum }\limits_{i=1}^{{c}_{1}+{c}_{2}}\log \left({P}_{\widehat{\lambda }}\left({W}_{o}={w}_{i}\right)\right)$$

10 $${\mathrm{LL}}_{{M}_{1}}=\mathop{\sum }\limits_{i=1}^{{c}_{1}}\log \left({P}_{{\widehat{\lambda }}_{c_1}}\left({W}_{o}={w}_{i}\right)\right)+\mathop{\sum }\limits_{i={c}_{1}+1}^{{c}_{1}+{c}_{2}}\log \left({P}_{{\widehat{\lambda }}_{c_2}}\left({W}_{o}={w}_{i}\right)\right)$$

where $\hat{\lambda }$ is the estimated latent insertion rate of the two groups of cells combined, and ${\widehat{\lambda }}_{1}$ and ${\widehat{\lambda }}_{2}$ are the estimated latent insertion rate for each group.

The likelihood ratio test statistic is defined by

11 $${\chi }^{2}=-2\left({\mathrm{LL}}_{{M}_{0}}-{\mathrm{LL}}_{{M}_{1}}\right)$$

with 1 d.f. We note that calculating the MLE for ssPoisson can be computer intensive and time consuming, given the distribution’s dependency on cell-specific capturing rate and peak width.

### Comparing insertion rate estimators through simulation

We used a Bernoulli model to simulate insertion locations with probability of insertion being *λ*/*L*_*p*_, where *λ* is a given insertion rate and *L*_*p*_ is the length of peak, specified as 500 bp in our simulation. For each insertion, we then simulated the primer configuration of the Tn5 dimer. Fragments with the viable primer configuration on both ends and with the amplifiable/mappable length were our final simulated PIC counts. With the counts, we used ssPoisson and Poisson distribution (after a twofold correction) to estimate the insertion rates across 500 cells. Examples of probability mass functions for Poisson and ssPoisson distributions are shown in Supplementary Fig. 8b.

### Type I error and power of different methods through simulation

We simulated a wide range of insertion rates, from 0.05 to 2.5 (per peak) for the evaluation. We simulated different level of log fold changes to be ±0.1, 0.15, 0.2 and 0.25 for the two groups. A total of 6,000 DAR peaks were generated with of combinations insertion rates and log fold change. The data were simulated under three settings:

1. 500 cells in each group, 5,000 non-DAR peaks and 6,000 DAR peaks with equal number of positive and negative log fold changes
2. 500 cells in one group and 200 cells in another group, 5,000 non-DAR peaks and 6,000 DAR peaks with equal number of positive and negative log fold changes
3. 500 cells in each group, 5,000 non-DAR peaks, 2,000 DAR peaks with positive log fold change and 4,000 DAR peaks with negative log fold changes

### Power comparison in real data

We used label permutation to establish a new null critical value for each method. Specifically, each cell received a random cell type label and DAR is conducted between the two groups using the new label. *P* values from the permutations were obtained and the fifth rank percentile was used as the correct critical value for each method. Since we do not know the true DAR set, we defined the pseudotrue DAR peaks as the union DAR set of the three methods, using their corresponding new critical values. Power for each method is then calculated by the number of DARs detected divided by the number of pseudotrue DARs.

### Reporting summary

Further information on research design is available in the Nature Portfolio Reporting Summary linked to this article.

## Online content

Any methods, additional references, Nature Portfolio reporting summaries, source data, extended data, supplementary information, acknowledgements, peer review information; details of author contributions and competing interests; and statements of data and code availability are available at 10.1038/s41592-023-02103-7.

### Supplementary information

Supplementary Information Supplementary Figs. 1–8 and Notes 1–4.

Reporting Summary

Supplementary Tables Supplementary Tables 1–6.

## Data Availability

All snATAC-seq datasets used in this study were obtained from public repositories with the following accession numbers: mouse kidney data^17^ (GEO accession number GSE157079), human cell line data^4^ (GEO accession number GSE162690), human BMMC data^28^ (GEO accession number GSE194122), mouse brain dscATAC-seq data^20^ (GEO accession number GSE123581), human brain scTHS-seq data^13^ (GEO accession number GSE97942), human adult sci-ATAC-seq data^30^ (GEO accession number GSE184462), human brain SNARE-seq2 data^22^ (Neuroscience Multi-omics Archive, RRID SCR_016152). We downloaded the 10x Genomics human PBMC data (including an snATAC-seq and an sn-multiome dataset^18^) from the 10x Genomics website (https://www.10xgenomics.com/resources/datasets). The list of enhancers in the blood sample was obtained from TRIPOD study^31^ (PMID 36055233), which include three queried databases: EnhancerAtlas v.2.0 (ref. ^32^, http://www.enhanceratlas.org), FANTOM5 (ref. ^33^, https://fantom.gsc.riken.jp/5/) and 4DGenome (ref. ^34^, https://bioinfo.vanderbilt.edu/AE/HACER/). We downloaded the GTEx whole blood eQTL summary statistics (v.8) from the GTEx Portal^35^ (dbGaP Accession phs000424.v8.p2).

## Code availability

All codes used in this project including the PIC algorithm are in the GitHub repository https://github.com/Zhen-Miao/PICsnATAC and Zenodo^36^.
