# Supplementary material for: Uniform quantification of single-nucleus ATAC-seq data with Paired-Insertion Counting (PIC) and a model-based insertion rate estimator
Source: Nat Methods. 2023 Dec 4;21(1):32–6. doi: 10.1038/s41592-023-02103-7 (PMC10776405; doi:10.1038/s41592-023-02103-7)
Supplement: Supplementary file 1 — Supplementary Figs. 1–8 and Notes 1–4. [file 41592_2023_2103_MOESM1_ESM.pdf]

nature methods

Brief Communication

https://doi.org/10.1038/s41592-023-02103-7

Uniform quantification of single-nucleus ATAC-seq data with Paired-Insertion Counting (PIC) and a model-based insertion rate estimator

In the format provided by the authors and unedited

Supplementary Materials:

Supplementary Table 1: Frequency of counts with different counting strategies (PBMC-5k data)
Supplementary Table 2: Summary of data quantification methods in real studies
Supplementary Table 3: Comparison between simulated and theoretical fragment counts for snATAC-seq data
Supplementary Table 4: Summary of datasets and several statistics included in this study
Supplementary Table 5: Correspondence between fragment-based and insertion-based strategies (kidney P0 data)
Supplementary Table 6: Linked peak-gene pairs in BMMC data unique to PIC and supported by public databases

Supplementary Note 1: Issues with insertion-based counting
Supplementary Note 2: Nucleosome density and peak substructure
Supplementary Note 3: Biological implications of DARs detected by PIC framework
Supplementary Note 4: Effects of different quantification approaches for downstream analysis

Supplementary Fig. 1-8

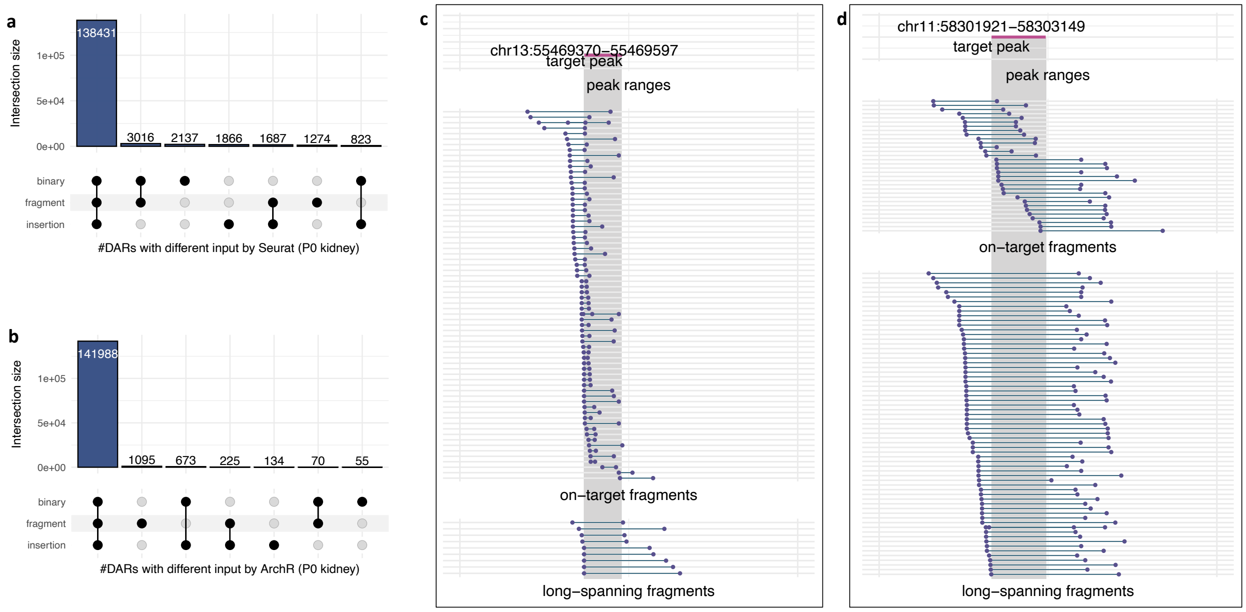

Supplementary Fig. 1.

(**a**) Number of significant Differentially Accessible Regions with different counting approaches using Seurat, for the P0 kindey dataset. (**b**) Number of significant Differentially Accessible Regions with different counting approaches using ArchR, for the P0 kindey dataset. (**c-d**) Two example peaks with inconsistent DAR results between the insertion and fragment counting strategies. Fragments were classified into on-target fragments, where both counting strategies output non-zero count and long-spanning fragments, where insertion-based counting outputs zero count but fragment-based counting outputs non-zero counts. An additional example is shown in **Fig. 1g**.

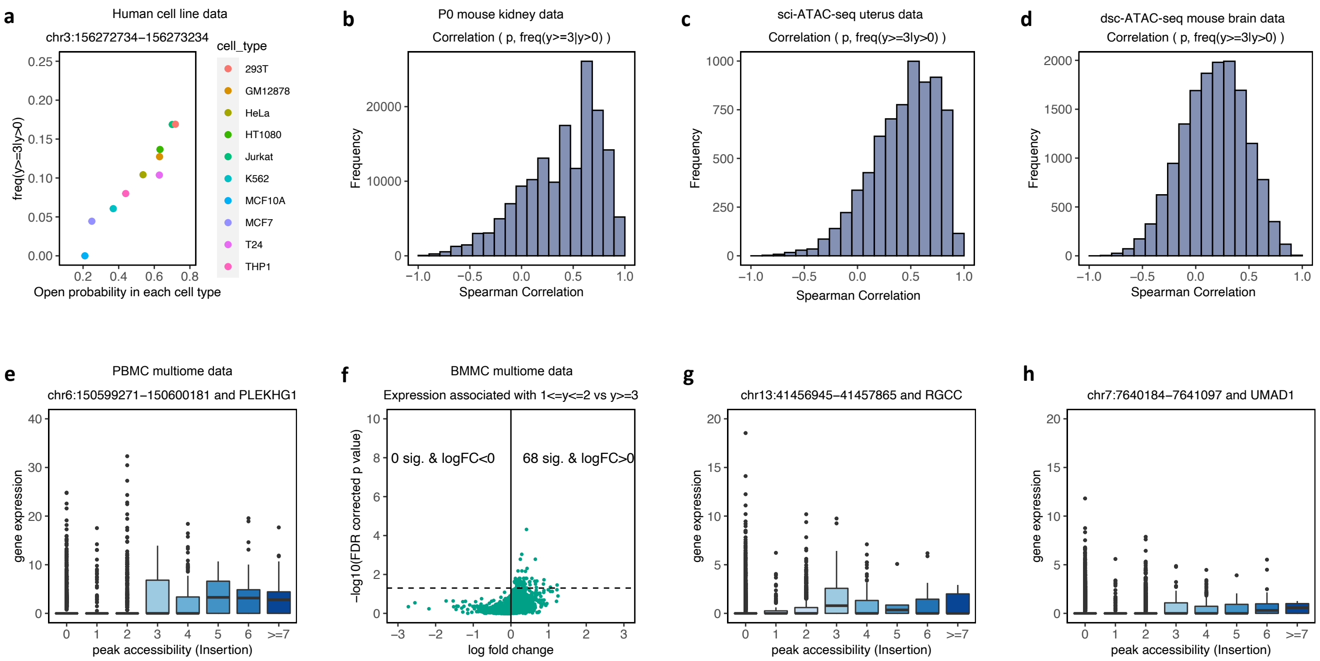

Supplementary Fig. 2.

(**a**) Example peak with correlated open probabilities and relative frequency of peaks with high-density insertion across cell types in the human cell line data. (**b-d**) Histogram of Spearman correlation coefficients between the probability of accessible peak in each group and the relative frequency of high-density insertion counts in P0 mouse kidney (**b**), sci-ATAC-seq uterus (**c**), and dsc-ATAC-seq mouse brain (**d**) data. (**e**) An example of peak-gene pair where gene expression levels are related to the TSS peak insertion counts in PBMC data. N=10,538 cells were examined over one independent experiment. For this panel and panel (**g-h**), center line in box plot represents median and the lower and upper hinges correspond to the first and third quartiles. The upper or lower whisker corresponds to 1.5 times the inter-quartile range or the largest/smallest values. (**f**) Volcano plot showing the normalized gene expression levels between cells with TSS peak insertion counts equal to 1 or 2 and cells with high-density TSS peak insertion counts in BMMC data. Two-sided Wilcoxon rank-sum test was used for the comparison and FDR correction was used to adjust for multiple comparisons. (**g-h**) Examples of peak-gene pairs where gene expression levels are related to the TSS peak insertion counts in BMMC data. N=6,740 cells were examined over one independent experiment.

a

	Motif	PWM	P value	Adjusted.p	Cell type
1	Elf4	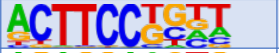	1e-785	<0.0001	CD14 Mono
2	PU.1	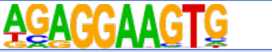	1e-773	<0.0001	CD14 Mono
3	IRF8	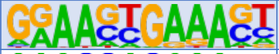	1e-470	<0.0001	CD14 Mono
4	RUNX1	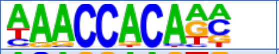	1e-209	<0.0001	CD14 Mono
5	ETS1	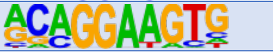	1e-648	<0.0001	CD4 T
6	BACH2	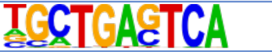	1e-83	<0.0001	CD4 T

b

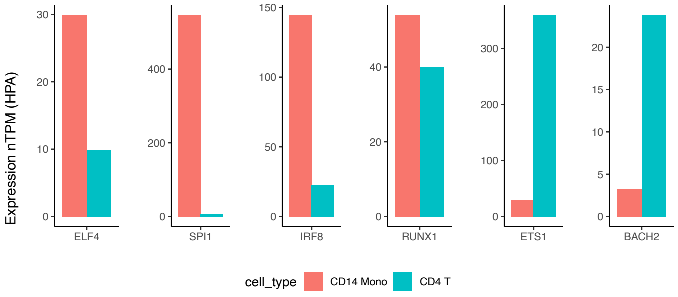

c

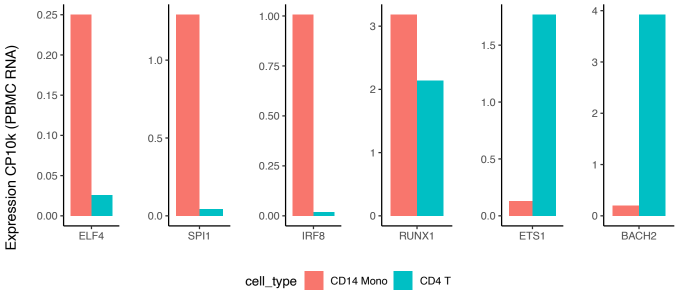

Supplementary Fig. 3.
(**a**) Summary of HOMER[1] motif enrichment results using the set of DARs identified uniquely by PIC framework, for the PBMC data. DAR is conducted between CD4+ Naïve T cells and CD14+ Monocytes. Enrichment p-values in HOMER was based on one-sided hypergeometric test. (**b-c**) The cell type-specific expression of corresponding TFs reported in motif enrichment, from the expression profile of the PBMC multiome data (**b**) or The Human Protein Atlas data (**c**).

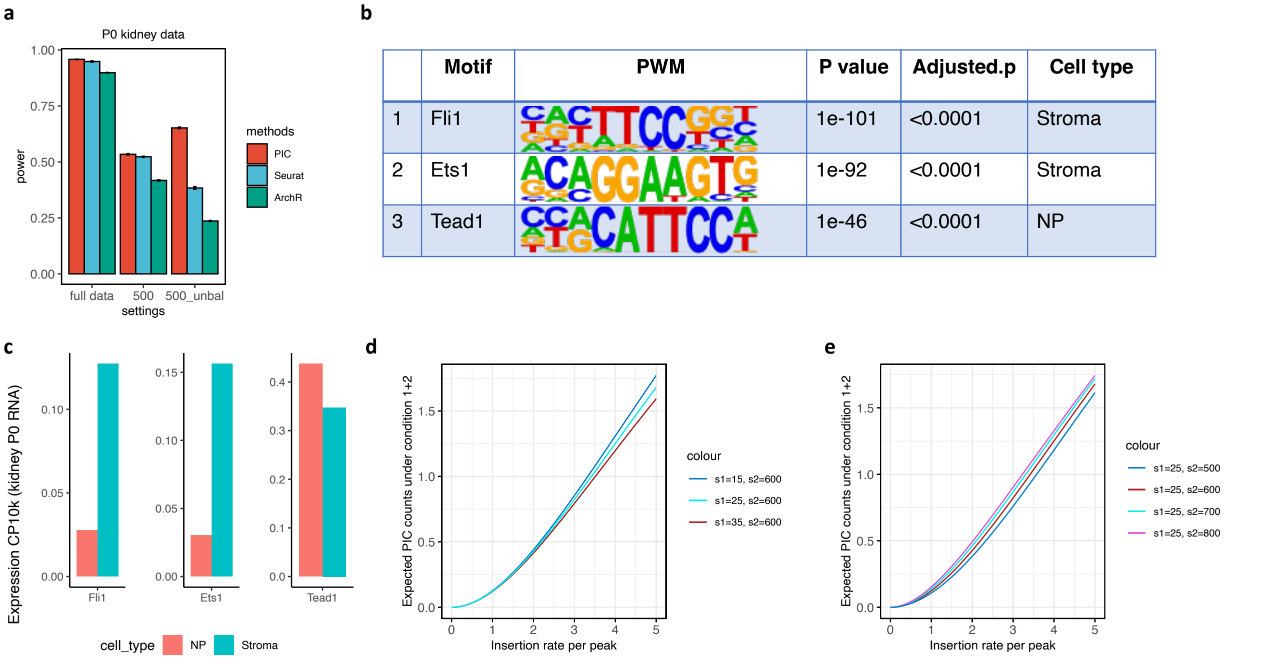

Supplementary Fig. 4.

(**a**) Power of PIC model, Seurat, and ArchR DAR tests under different settings with P0 kidney data. 500_unbal represents the condition when 500 cells in each group is sampled, but with different mean capturing rate. (**b**) Summary of HOMER motif enrichment results using the set of DARs identified uniquely by PIC framework, for the P0 kidney data. DAR is conducted between Stroma cells and Nephron Progenitor (NP) cells. Enrichment p-values in HOMER was based on one-sided hypergeometric test. (**c**) The cell type-specific expression of corresponding TFs reported in motif enrichment, from a separate P0 kidney scRNA-seq data. (**d-e**) The effect of different s1 and s2 values on the expectation values of ssPoisson distribution.

a

b

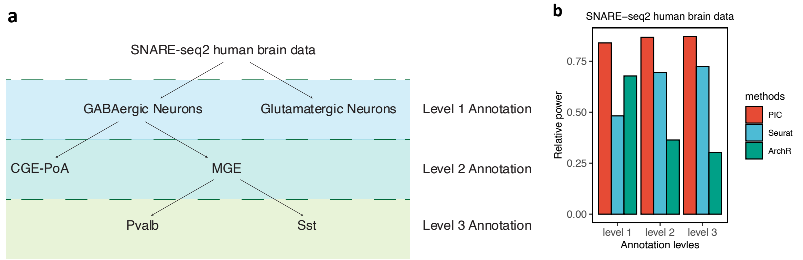

c

	Level 1	Level 2	Level 3
PIC	17647	3290	1164
Seurat	10126	2634	967
ArchR	14240	1379	404

Supplementary Fig. 5.

(**a**) Three annotation levels in SNARE-seq2 human brain data that are used in evaluating DAR performance of PIC, Seurat, and ArchR methods. (**b**) Power of PIC model, Seurat, and ArchR DAR tests at different annotation levels of the SNARE-seq2 human brain data. (**c**) Number of detected significant DARs at different annotation levels for the three methods.

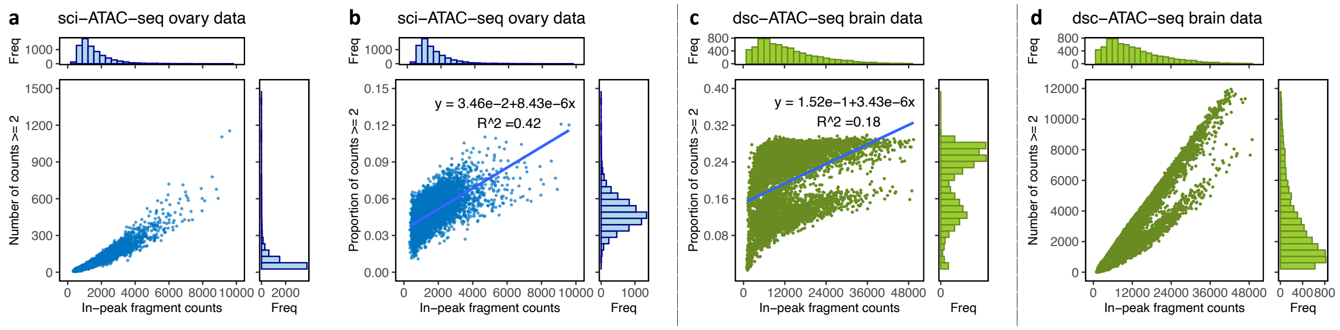

Supplementary Fig. 6.

(**a-b**) The relationship between in-peak fragment counts and number (**a**) / proportion (**b**) of counts greater than or equal to two for each single cell in the sci-ATAC-seq ovary dataset. (**c-d**) The relationship between in-peak fragment counts and number (**c**) / proportion (**d**) of counts greater than or equal to two for each single cell in the dsc-ATAC-seq brain dataset.

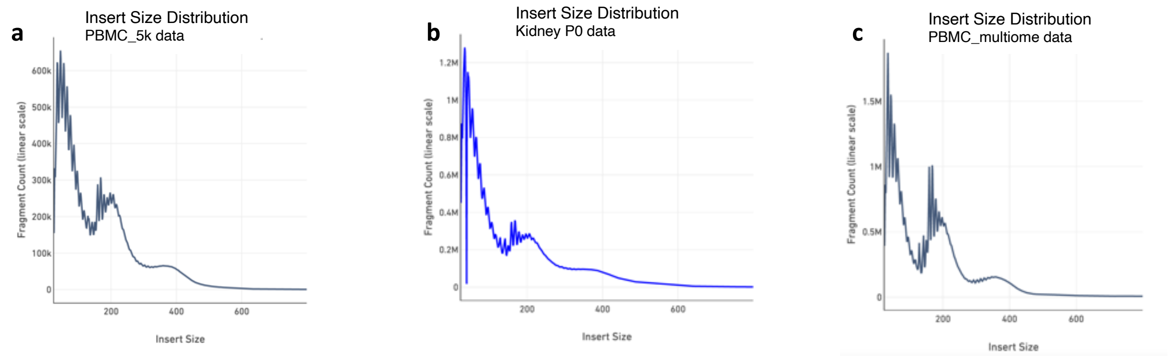

Supplementary Fig. 7.

(**a**) Tn5 Insert size distribution in 10X Genomics PBMC-5k snATAC-seq dataset. (**b**) Tn5 Insert size distribution in P0 mouse kidney snATAC-seq dataset. (**c**) Tn5 Insert size distribution in 10X Genomics PBMC-10k Multiome dataset.

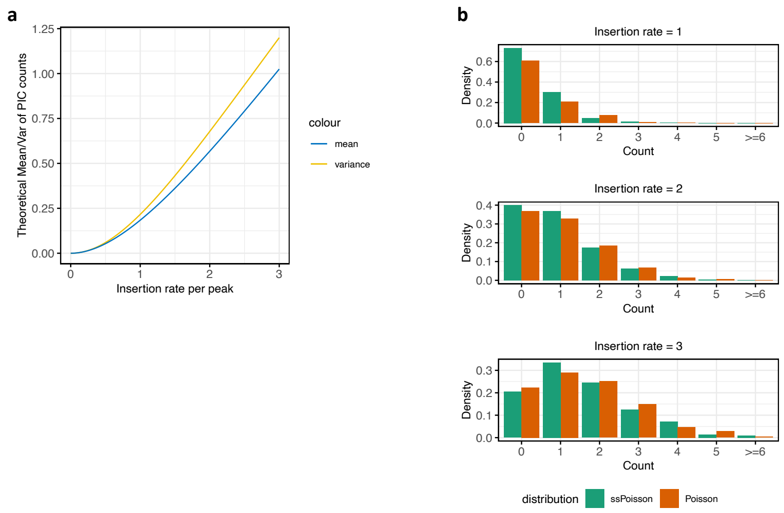

Supplementary Fig. 8.

(**a**) Theoretical mean and variance of PIC counts under different insertion rates per peak, under the distribution with constraint of correct primer orientation. (**b**) Comparing the probability mass function (PMF) between Poisson and ssPoisson distribution for different underlying insertion rates.

Supplementary Note 1. Issues with insertion-based counting

In the standard ATAC-seq experiments, Tn5 transposase dimers function by inserting adapters into open chromatin regions, which causes a break in the corresponding locus. When two adjacent Tn5 tagmentation events take place with the correct primer configuration (one forward and one reverse primer, see ref.[2]), a viable fragment is formed for PCR amplification and sequencing. In general, four insertions with the right orientations are needed to generate two fragments. However, three adjacent Tn5 tagmentation events with the correct primer configuration can also result in two viable fragments. Here we briefly describe the insertion-based counting algorithm implemented in ArchR[3] (v.1.0.2), and then we discuss two issues with the current insertion-based counting methods.

To construct the insertion count-by-cell matrix, ArchR (https://github.com/GreenleafLab/ArchR) obtains the offset-adjusted fragments from snATAC-seq pre-processing. Each fragment is then processed independently in parallel. The two insertion loci, denoted as the "left" and "right" insertion, are each mapped to the peak regions by using the "findOverlaps" function in GenomicRanges package[4]. A sparse matrix is then constructed by pulling together the "left" and "right" insertions in each cell. The related source codes are available at the ArchR GitHub repository (https://github.com/GreenleafLab/ArchR/blob/master/R/MatrixFeatures.R).

The first issue with this insertion-based counting originates from the fact that it disregards the configuration of adjacent fragments. More specifically, as we described, the existing approaches consider each fragment independently, ignoring the possibility of shared insertion sites between fragments. Thus, when there are two fragments with only three insertion events, insertion-based counting will count it as four instead (e.g., cell 3 in **Fig. 1a**). In the real snATAC-seq datasets, it is not uncommon for fragments to share an insertion locus. For instance, in the PBMC multiome data, more than 20% adjacent fragments have shred insertion locus (**Supplementary Fig. 2h**), so such an issue cannot be ignored.

The second issue with the insertion-based counting is due to the events where one insertion locus is outside of the peak/bin boundary (e.g., cell 1 in **Fig. 1a**). Consequently, the counted number of insertions can be smaller than the actual insertions and is very sensitive to the peak/bin boundary. It is clearly problematic for fixed-sized bins as features since the bin boundaries are arbitrary. We also show below that even for

peaks with boundaries defined by data-driven approaches, the insertion counts do not have a clear biological meaning compared with fragment-based counting methods that we discussed in the main text.

To examine whether the insertion count of one or two represent different signals, we firstly calculated the proportion of cells with counts equal to two given counts being either one or two (i.e., $P(y = 2|y = 1\ or\ 2)$) for each cell type in the human cell line data. We then computed its correlation with the open probability for the same cell type (see **Methods** for open probability definition and estimation). We observed a symmetric distribution of Spearman correlation coefficients centered around 0 (**Supplementary Note Fig. 1a**), with only ~0.08% peaks showing significant correlations (FDR corrected p value < 0.05). This indicates that there is essentially no biological implication of observing a count of two vs one in terms of the actual chromatin open probability. Notably, the same analysis on higher density counts (counts greater than two, i.e., at least two fragments) shows largely positive correlation coefficients (**Fig. 2a**, see **Results**), in contrast to this result. Similar results are observed with the mouse kidney data and the sci-ATAC-seq data (**Supplementary Note Fig. 1b-c**). Two example peaks with cell type-specific open probability and proportion of observing one vs two are shown, and there was no clear correlation (**Supplementary Note Fig. 1d-e**). In contrast, for the same examples, the proportion of high-density counts are positively correlated with the open probability (**Fig. 2b** and **Supplementary Fig. 2a**).

As a second analysis, we checked whether the count of one vs two in TSS peak indicates differences in the corresponding gene expression. Using the PBMC multiome data with joint RNA and ATAC modality from the same cell, we compared gene expression levels associated with TSS peak insertion count = 1 against those with count = 2. We found only 18 significant peak-gene pairs after FDR correction, nine of which have positive log fold change (**Supplementary Note Fig. 1f**). As a contrast, when we compare insertion count one or two versus higher density counts, we found 199 significant peak-gene pairs and 189 of them had positive log-fold changes, suggesting the true difference (**Fig. 2c**). In addition, among all the peak-gene pairs (including non-significant ones), 52% showed higher expression in the group with count = 2, again suggesting no increased gene expression with the count two. We also analyzed a BMMC dataset, and we obtained a consistent result (**Supplementary Note Fig. 1g**).

In sum, here we showed two issues with the current insertion-based counting strategy, and our analyses with real dataset also indicated that there was no significant biological information difference between the

observed count of two versus one for insertion-based counting strategy but important information in higher number of insertions.

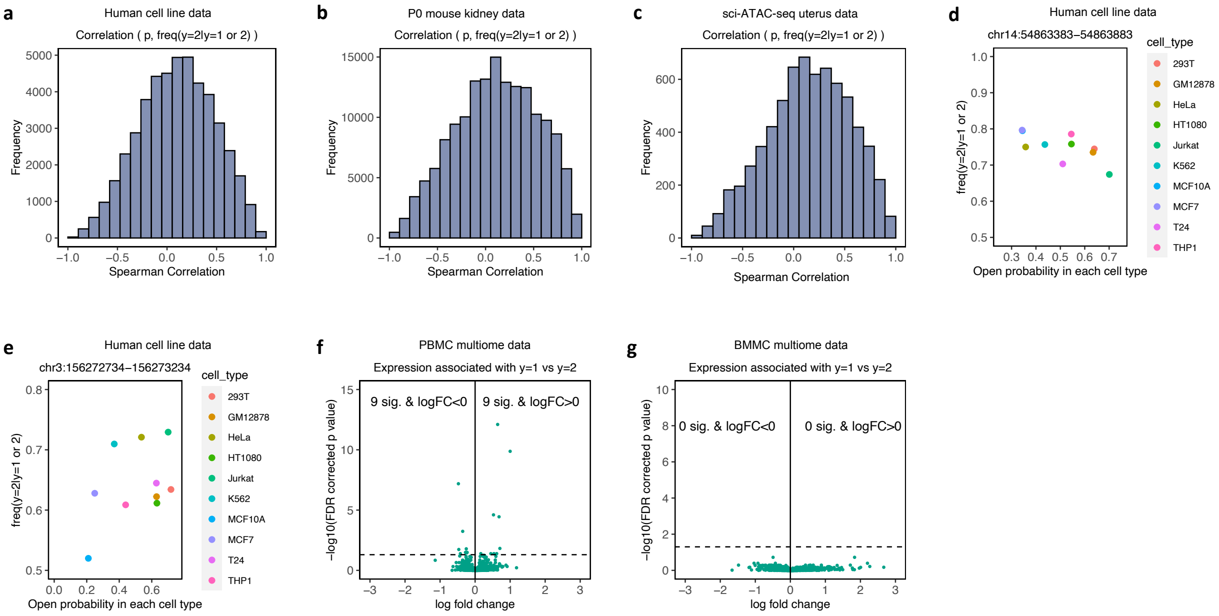

Supplementary Note Fig. 1.

(**a-c**) Histogram of Spearman correlation coefficients between open probability in each group and the relative frequency of counts equal to 2 over counts being either 1 or 2 in human cell line data (**a**), P0 mouse kidney data (**b**), and sci-ATAC-seq uterus data (**c**). (**d-e**) Relationship between the open probabilities and the relative frequency of counts equal to 2 for two example peaks in human cell line data. Another example was displayed in **Supplementary Fig. 1b-c**. (**f-g**) Volcano plot showing the normalized gene expression levels between cells with TSS peak insertion counts equal to 1 and 2 in PBMC data (**f**) or BMMC data (**g**). In the PBMC data, N=10,538 cells were examined over one independent experiment, and in the BMMC data, N=6,740 cells were examined over one independent experiment.

Supplementary Note 2. Nucleosome density and peak substructure

The datasets we examined were based on peaks identified by Cell Ranger ATAC or MACS2 (narrow peak setting), resulting in median peak width of 446 and 876, respectively (see **Supplementary Note Fig. 2a**). SPP[5,6], another common peak-calling approach, and ArchR[3] "TileMatrix" generate fixed-width peaks, usually specified to 500 bp. Some methods resize peak to a uniform width of 500 bp centered at the peak summit[7,8]. Although regulatory elements vary in length, various studies suggest typical regulatory regions are around several hundred base pairs[9], with the median length of predicted enhancer chromatin state ~600 bp[10]. Thus, most peak widths are around the typical length of one cis-regulatory element.

For a given region of interest (i.e., peak), it is possible that there might be multiple CREs that can be independently accessible and create multiple ATAC-seq fragements. However, we noticed that fragments with shared insertion site are common, suggesting against the model of multiple independent elements. To assess the possibility of multiple CREs using the PBMC multiome data, we assessed within each cell, the distance between adjacent fragments (limited to < 1000 bp spacing). The majority of fragments that locate in the same peak are close to each other (75% fragments are within 200 bp of one another), and in particular, 22.6% adjacent peaks have shared insertion site (distance = 1, **Supplementary Note Fig. 2b**). As an example, we examined the observed fragments around *CD74* gene (**Supplementary Note Fig. 2c**). We do not observe bimodal insertion patterns, but rather relatively continuous insertion loci. In addition, by resizing the TSS peaks in **Fig. 2c** to equal 500 bp, there are still 126 significant peak-gene pairs with positive log fold change (**Supplementary Note Fig. 2d**). Taken together, empirical data seem to suggest that the quantitative aspect of the scATAC-seq data can be ascribed to differential local insertion rate at a single CRE.

While not common, it is still possible that the quantitative property of snATAC-seq count may arise from multiple sub-regulatory regions in one peak. Through multi-scale footprinting[11], researchers have identified substructures of peak regions, which they call "sub cis-regulatory regions" or sub-CREs. These CREs may represent a nucleosome or nucleosome-free regions in between. It has been shown that for some peaks, sub-CRE accessibility and gene expression correlation is higher than that between CRE (peak) accessibility and gene expression, suggesting that these sub-CREs are functional units, whose signals are masked at the CRE level. If the CREs act in an additive manner for gene regulation, simply treating the peak counts as quantitative measure will not be affected by sub-CRE structure. On the other hand, if the

sub-CREs act in a non-additive manner (e.g., by mutual antagonism), there will be a more compliczted relationship between ATAC-seq fragements and regulatory status. Future research is needed to understand to what extend quantitative information is due to multiple sub-regulatory elements harbored within one peak.

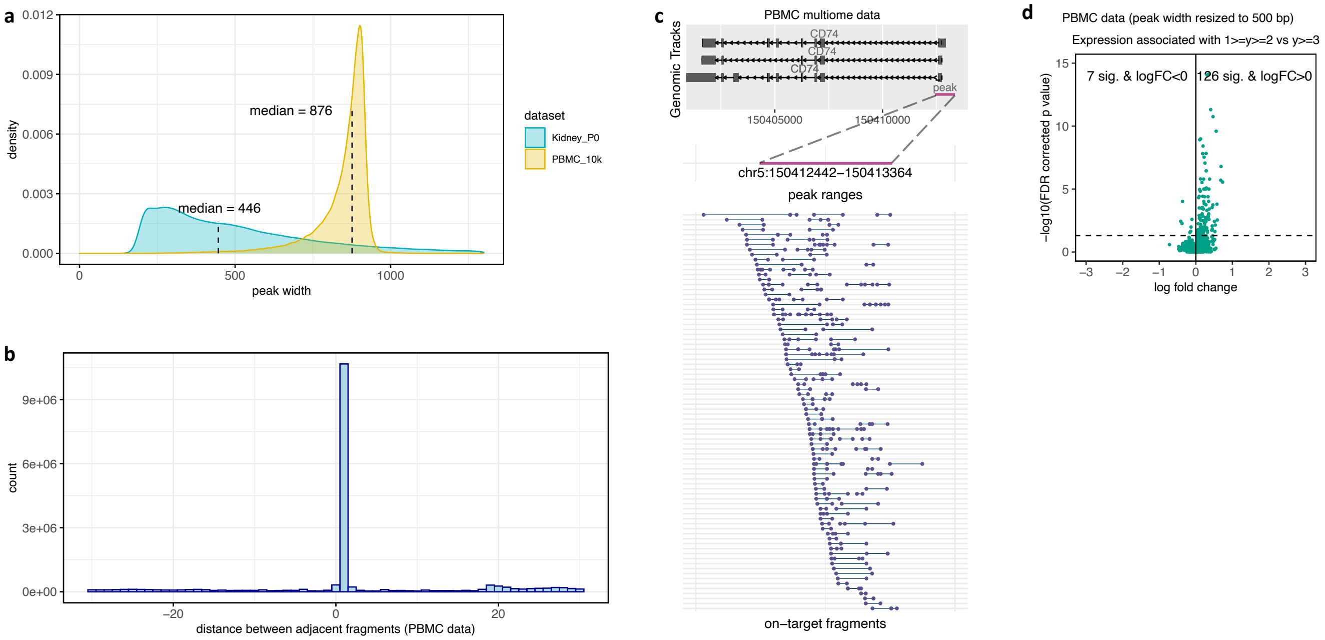

Supplementary Note Fig. 2.

(**a**) Distribution of peak width for P0 mouse kidney and PBMC multiome data. The peak calling approach is MACS2 for P0 mouse kidney data and is Cell Ranger ATAC for PBMC multiome data. (**b**) Distribution of distance between adjacent fragments (distance within 1000 bp). The peak is when distance = 1, i.e., when the fragments have shared insertion locus. (**c**) An example insertion-fragment landscape around the CD74 promoter peak, where gene expression is significantly correlated with the promoter ATAC fragment counts. Upper panel: genomic track of CD74 genes, the longest three isoforms were shown. Lower panel: the zoomed in peak ranges and corresponding insertion-fragment patterns for different cells. (**d**) Volcano plot showing the normalized gene expression levels between cells with TSS peak insertion counts equal to 1 or 2 and cells with high-density TSS peak insertion counts in PBMC data (after resizing all peaks to 500 bp). Two-sided Wilcoxon rank-sum test was used for the comparison and FDR correction was used to adjust for multiple comparisons.

Supplementary Note 3. Biological implications of DARs detected by PIC framework

To determine the biological implications of the set of DARs that are detected by PIC model but not detected with existing methods, we conducted motif enrichment analysis with HOMER[1]. Among the PIC-unique DARs for the PBMC data, we were able to identify the enriched motifs ELF4, PU.1 (*SPI1* gene), IRF8, and RUNX1 in monocytes, and ETS1 and BACH2 in T cells (**Supplementary Fig. 3a**). The corresponding cell type-specific expression of the inferred transcription factor (TF) were confirmed in the multiomics gene expression of the same data and public single cell expression data from The Human Protein Atlas[12] (**Supplementary Fig. 3b-c**). Note that this analysis is only comparing two cell types, so the identified enrichment only suggests a higher signal in one cell type than the other (as opposed to “cell type markers”). We also analyzed the mouse kidney P0 dataset with lower sequencing depth (less quantitative information), where we compared the DNA accessibility landscape between stroma and nephron progenitors and our approach remains the most powerful choice, with 1% and 6% increase in power for the full data, and 72.5% and 183.2% increase in power for the unbalanced subsampled data compared with Seurat and ArchR, respectively (**Supplementary Fig. 4a**). We were able to identify subtle enrichment of Fli1 and Ets1 in stroma, and Tead1 in nephron progenitors (**Supplementary Fig. 4b**). These weak signals were masked by other methods but were identified with the more sensitive PIC framework. We confirmed these cell type differential signals with the gene expression profile in an (unmatched) scRNA-seq dataset (**Supplementary Fig. 4c**).

We next analyzed a human brain data from the SNARE-seq2 protocol[13]. In this dataset, cells were grouped at three different annotation levels: at the first level, cells were categorized into GABAergic neurons, Glutamatergic neurons, and non-neuronal cells; at the second and third levels, each category was then subdivided into sub-cell types (**Supplementary Fig. 5a**). We sought to compare the performance of DAR methods at different level of cell annotations (thus, different level of cell similarities). For each level, we performed pairwise comparisons on randomly sampled 1000 cells within each group. Using the same procedure as described above, our findings consistently demonstrate that the PIC model outperforms other alternatives in detecting DARs (**Supplementary Fig. 5b-c**), where PIC shows an average increase of 39.7% and 116.9% in the number of DARs compared with Seurat and ArchR, respectively.

Supplementary Note 4. Effects of different quantification approaches for downstream analysis

We have shown that quantitative counts in snATAC-seq data contain biologically relevant information. Here, we explore examples of how incorporating quantitative information will affect downstream biological inferences for models of regulatory interactions and association between chromatin states and gene expression. As we noted above, to be more broadly applicable and to incorporate only direct evidence, we believe ATAC-seq counts should be based on insertion events. Therefore, we compare standard insertion counting, binary coarse-graining, and PIC method for generating the input data matrix.

To evaluate how the input matrix affects gene activity score inference, we analyzed the multiome BMMC data[14] with expert-annotated cell type labels, and the PBMC data with metacell inferred in ref.[15]. We constructed PIC count matrix, insertion count matrix, and binarized matrix for the BMMC and PBMC data, and used three methods to infer gene activity score: sum of accessibility[16] ("sum acc"), exponential-decay weighted sum of accessibility (MAESTRO[17]), and LASSO on accessibility[18] (peak LASSO). "Sum acc" is the vanilla method where the gene activity is inferred by summing up the accessibility of peaks in proximity to genic regions. MAESTRO assumes an exponentially decayed peak weights based on distance to TSS, which showed increased performance than the "sum acc" method. We used the "enhanced" inference framework and set maximum distance to 1,000 for the promoter model and 10,000 for the enhancer model, respectively (for details of these parameters see[17]). The "peak LASSO" method requires a training set of matched multiome data, and it employs a LASSO regression of gene expression by the peak accessibility in the proximity to retain a sparse set of predictive peaks and their coefficients. Then these coefficients are used to predict expression for new datasets. We used leave-one-out strategy to obtain the predicted activity scores.

For each cell type, we calculated the Spearman correlation coefficients between gene expression and gene activity scores among highly variable genes. Briefly, we summed up all expression/activity values within the same cell type (for BMMC data) or metacell (for PBMC data) and then normalized by overall sequencing depth for both matrices. Genes were filtered by taking the top 5,000 highly variable genes in expression matrix followed by requiring more than 50 cells showing non-zero gene activities. We found that the average correlation is significantly higher with PIC matrix compared with binary or insertion inputs, across various settings (**Supplementary Note Fig. 3a-b**). We note that the insertion-based

counting was out-performed by binary matrix, suggesting that the inaccurate counting may introduce false signals. The overall low performance of the peak LASSO model for BMMC data indicated that the small number of cell types (n=21) are not sufficient for this regression-based method to be learning meaningful coefficients for peaks. While there were significant average differences, over all genes, the difference in the correlation coefficient was small, which is not surprising since a large percent of the peaks will have same counts (zero or one) regardless of the method. We suggest that when many peaks are part of the model predicting gene expression, coarse-graining to binary states or errors of insertion counting at individual peaks can be compensated by the multi-peak optimization of the gene activity models.

We next explored the impact of the type of input matrix on the inference of peak-gene interactions for gene expression regulation. We followed the methods in SHARE-seq[19] to conduct a peak-gene correlation analysis on the BMMC data, which aims to identify accessible peaks and gene expression that were significantly correlated by comparing them with background peak-gene pairs. The correlation coefficient is compared with a set of background peaks that match the GC content, accessibility, and length of the peak. The RNA expression and peak accessibility are smoothed by neighborhood to resolve the sparsity of the data, as reported in Cell Ranger ARC[20]. For each gene, the tests are conducted between peaks within 10,000 bp of gene body, and Pearson Correlation coefficient is used for correlation analysis. Peak-gene pairs with p-value < 0.05 were retained as significant pairs. We were able to identify 6860 significant peak-gene pairs with PIC input, representing a 5.7% and 5.2% increase compared with binary or insertion input, respectively. More precisely, we identified 1029 peak-gene pairs that were exclusively detected with PIC. To verify the validity of these peak-gene pairs, we cross-referenced them with four public databases that contain experimental assays to detect putative peak-gene associations, as previously reported in[15]. These databases include three enhancer atlas (FANTOM5, 4DGenome, EnhancerAtlas2.0) and one eQTL atlas (GTEx), focusing on the data derived from blood cells lines or whole blood samples. Out of the 1029 PIC-unique peak-gene pairs, 469 pairs were supported by at least one database. In contrast, the sets of peak-gene pairs unique to binary or insertion input were smaller, consisting of 353 and 348 total pairs, with 188 and 137 pairs supported by existing database, respectively. The list of identified peak-gene pairs that are supported by existing database were summarized in **Supplementary Table 6**.

The effects of data quantification depend both on the degree of quantitative information in the data, typically governed by the sequencing depth and experimental design, and the effectiveness of the existing

computational methods in incorporating quantitative information, regardless of the counting method. To evaluate the level of quantitative information in each dataset, we defined a quantity called “proportion of high-density peaks”. For each peak, we calculate the number of cells with PIC counts greater than or equal to two, and the number of cells with non-zero PIC counts and divided the two values to obtain the “proportion of high-density peaks”. In **Supplementary Note Fig. 3**, we show the single cell distribution of high-density proportions across each peak for five representative datasets generated with sci-ATAC-seq, 10X Genomics snATAC-seq, dscATAC-seq, and s3-ATAC-seq, and summarize the global high-density count proportion in **Supplementary Table 4**. Data from sci-ATAC-seq have overall low coverage, and most peaks do not have high-density count. By contrast, data from s3-ATAC-seq show stronger quantitative information, with ~99% peaks contain more than 50% high-density counts. With future technology development, accurate quantification will be vital for extracting biological information.

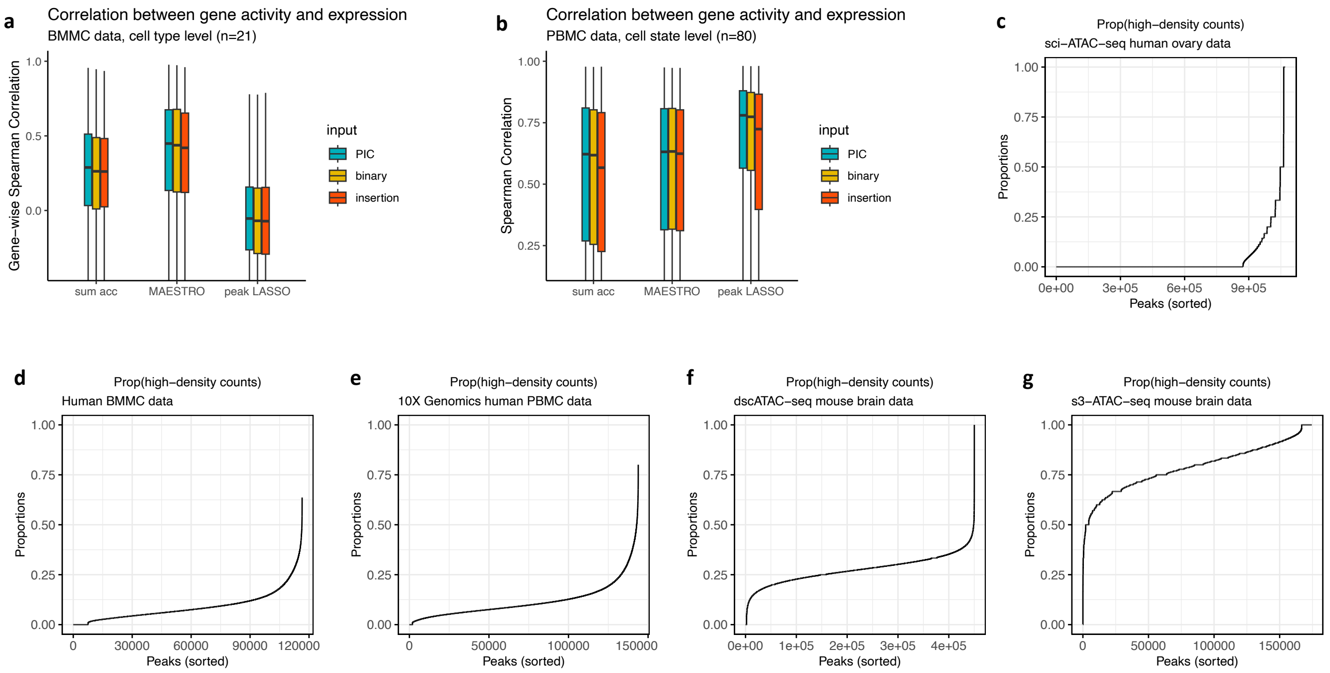

Supplementary Note Fig. 3.
(**a-b**) Spearman correlation coefficients between gene expression and gene activity scores across cell groups for BMMC (**a**) and PBMC (**b**) data with insertion matrix, binary matrix, or PIC matrix as input. Three activity score inference methods are used, sum acc represents the “sum of accessibility” method. Center line in box plot represents median and the lower and upper hinges correspond to the first and third quartiles. The upper or lower whisker corresponds to 1.5 times the inter-quartile range or the largest/smallest values. (**c-g**) Proportion of high density counts for each peak in five datasets: sci-ATAC-seq human ovary data (**c**), 10X Genomics human BMMC data (**d**), 10X Genomics human PBMC data (**e**), dscATAC-seq mouse brain data (**f**), and s3-ATAC-seq mouse brain data (**g**).

Supplementary Note References:

1. Heinz, S. *et al.* Simple Combinations of Lineage-Determining Transcription Factors Prime cis-Regulatory Elements Required for Macrophage and B Cell Identities. *Mol. Cell* **38**, 576–589 (2010).
2. Adey, A. C. Tagmentation-based single-cell genomics. *Genome Res.* **31**, 1693–1705 (2021).
3. Granja, J. M. *et al.* ArchR is a scalable software package for integrative single-cell chromatin accessibility analysis. *Nat. Genet.* **53**, 403–411 (2021).
4. Lawrence, M. *et al.* Software for Computing and Annotating Genomic Ranges. *PLOS Comput. Biol.* **9**, e1003118 (2013).
5. Kharchenko, P. V., Tolstorukov, M. Y. & Park, P. J. Design and analysis of ChIP-seq experiments for DNA-binding proteins. *Nat. Biotechnol.* **26**, 1351–1359 (2008).
6. Lake, B. B. *et al.* Integrative single-cell analysis of transcriptional and epigenetic states in the human adult brain. *Nat. Biotechnol.* **36**, 70–80 (2018).
7. Schep, A. N., Wu, B., Buenrostro, J. D. & Greenleaf, W. J. chromVAR: inferring transcription-factor-associated accessibility from single-cell epigenomic data. *Nat. Methods* **14**, 975–978 (2017).
8. Zhang, K. *et al.* A single-cell atlas of chromatin accessibility in the human genome. *Cell* **184**, 5985-6001.e19 (2021).
9. Gasperini, M., Tome, J. M. & Shendure, J. Towards a comprehensive catalogue of validated and target-linked human enhancers. *Nat. Rev. Genet.* **21**, 292–310 (2020).
10. Kellis, M. *et al.* Defining functional DNA elements in the human genome. *Proc. Natl. Acad. Sci.* **111**, 6131–6138 (2014).
11. Hu, Y. *et al. Single-cell multi-scale footprinting reveals the modular organization of DNA regulatory elements*. http://biorxiv.org/lookup/doi/10.1101/2023.03.28.533945 (2023) doi:10.1101/2023.03.28.533945.

12. Karlsson, M. *et al.* A single–cell type transcriptomics map of human tissues. *Sci. Adv.* **7**, eabh2169 (2021).

13. Bakken, T. E. *et al.* Comparative cellular analysis of motor cortex in human, marmoset and mouse. *Nature* **598**, 111–119 (2021).

14. Luecken, M. *et al.* A sandbox for prediction and integration of DNA, RNA, and proteins in single cells. in *Proceedings of the Neural Information Processing Systems Track on Datasets and Benchmarks* (eds. Vanschoren, J. & Yeung, S.) vol. 1 (2021).

15. Jiang, Y. *et al.* Nonparametric single-cell multiomic characterization of trio relationships between transcription factors, target genes, and cis-regulatory regions. *Cell Syst.* **13**, 737-751.e4 (2022).

16. Stuart, T., Srivastava, A., Madad, S., Lareau, C. A. & Satija, R. Single-cell chromatin state analysis with Signac. *Nat. Methods* **18**, 1333–1341 (2021).

17. Wang, C. *et al.* Integrative analyses of single-cell transcriptome and regulome using MAESTRO. *Genome Biol.* **21**, 198 (2020).

18. Cao, J. *et al.* Joint profiling of chromatin accessibility and gene expression in thousands of single cells. *Science* **361**, 1380–1385 (2018).

19. Ma, S. *et al.* *Chromatin potential identified by shared single cell profiling of RNA and chromatin*. http://biorxiv.org/lookup/doi/10.1101/2020.06.17.156943 (2020) doi:10.1101/2020.06.17.156943.

20. Algorithms for computation of feature linkages -Software -Single Cell Multiome ATAC + Gene Exp. -Official 10x Genomics Support. https://support.10xgenomics.com/single-cell-multiome-atac-gex/software/pipelines/latest/algorithms/feature-linkage.
