# Supplementary material for: Uniform quantification of single-nucleus ATAC-seq data with Paired-Insertion Counting (PIC) and a model-based insertion rate estimator
Source: Nat Methods. 2023 Dec 4;21(1):32–6. doi: 10.1038/s41592-023-02103-7 (PMC10776405; doi:10.1038/s41592-023-02103-7)
Supplement: Supplementary file 2 — Reporting Summary [file 41592_2023_2103_MOESM2_ESM.pdf]

Corresponding author(s): Junhyong Kim, Ph.D.

Last updated by author(s): Jun 5, 2023

Reporting Summary

Nature Portfolio wishes to improve the reproducibility of the work that we publish. This form provides structure for consistency and transparency in reporting. For further information on Nature Portfolio policies, see our Editorial Policies and the Editorial Policy Checklist.

Statistics

For all statistical analyses, confirm that the following items are present in the figure legend, table legend, main text, or Methods section.

n/a	Confirmed	
☐	☒	The exact sample size (*n*) for each experimental group/condition, given as a discrete number and unit of measurement
☐	☒	A statement on whether measurements were taken from distinct samples or whether the same sample was measured repeatedly
☐	☒	The statistical test(s) used AND whether they are one- or two-sided<br>*Only common tests should be described solely by name; describe more complex techniques in the Methods section.*
☐	☒	A description of all covariates tested
☐	☒	A description of any assumptions or corrections, such as tests of normality and adjustment for multiple comparisons
☐	☒	A full description of the statistical parameters including central tendency (e.g. means) or other basic estimates (e.g. regression coefficient) AND variation (e.g. standard deviation) or associated estimates of uncertainty (e.g. confidence intervals)
☐	☒	For null hypothesis testing, the test statistic (e.g. *F*, *t*, *r*) with confidence intervals, effect sizes, degrees of freedom and *P* value noted<br>*Give P values as exact values whenever suitable.*
☒	☐	For Bayesian analysis, information on the choice of priors and Markov chain Monte Carlo settings
☒	☐	For hierarchical and complex designs, identification of the appropriate level for tests and full reporting of outcomes
☐	☒	Estimates of effect sizes (e.g. Cohen's *d*, Pearson's *r*), indicating how they were calculated

Our web collection on statistics for biologists contains articles on many of the points above.

Software and code

Policy information about availability of computer code

Data collection	No Software was used for data collection.
Data analysis	We used R v 4.2.3 for the data analysis, with the following R packages: data.table v 1.14.8, GenomicRanges v 1.50.2, IRanges v 2.32.0, parallel v 4.2.3, Rsamtools v 2.14.0, dplyr v 1.1.2, Rcpp v 1.0.10, stringr v 1.5.0, Seurat v 4.3.0, presto_1.0.0, ArchR v 1.0.2, Cicero v 1.17.2<br><br>We used the following softwares: HOMER v 4.10.4, IGV v 2.8.9, Cell Ranger ATAC v 2.0.0, Cell Ranger ARC v 2.0.0<br><br>The custom codes and algorithms developed in this study are available at GitHub (https://github.com/Zhen-Miao/PIC-snATAC).

For manuscripts utilizing custom algorithms or software that are central to the research but not yet described in published literature, software must be made available to editors and reviewers. We strongly encourage code deposition in a community repository (e.g. GitHub). See the Nature Portfolio guidelines for submitting code & software for further information.

Data

Policy information about availability of data

All manuscripts must include a data availability statement. This statement should provide the following information, where applicable:

- Accession codes, unique identifiers, or web links for publicly available datasets
- A description of any restrictions on data availability
- For clinical datasets or third party data, please ensure that the statement adheres to our policy

All data used in this study were from public datasets. We downloaded the following snATAC-seq datasets from public repositories: mouse kidney data (GEO accession number GSE157079), human cell line data (GEO accession number GSE162690), human BMMC data (GEO accession number GSE194122), mouse brain dscATAC-seq data (GEO accession number GSE123581), human brain scTHS-seq data (GEO accession number GSE97942), human adult sci-ATAC-seq data (GEO accession number GSE184462), human brain SNARE-seq2 data (Neuroscience Multi-omics Archive, RRID SCR_016152). We downloaded the 10X Genomics human PBMC data (including a snATAC-seq and a sn-multiome dataset) from the 10X Genomics website (https://www.10xgenomics.com/resources/datasets). The list of enhancers in the blood sample was obtained from TRIPOD study (PMID 36055233), which include three quired databases: EnhancerAtlas2.0, (http://www.enhanceratlas.org), FANTOM5 (https://fantom.gsc.riken.jp/5/), and 4DGenome, (https://bioinfo.vanderbilt.edu/AE/HACER/). We downloaded the GTEx whole blood eQTL summary statistics (V8) from the GTEx Portal (dbGaP Accession phs000424.v8.p2).

Human research participants

Policy information about studies involving human research participants and Sex and Gender in Research.

Reporting on sex and gender	This study does not involve human research participants
Population characteristics	This study does not involve human research participants
Recruitment	This study does not involve human research participants
Ethics oversight	This study does not involve human research participants

Note that full information on the approval of the study protocol must also be provided in the manuscript.

Field-specific reporting

Please select the one below that is the best fit for your research. If you are not sure, read the appropriate sections before making your selection.

☒ Life sciences ☐ Behavioural & social sciences ☐ Ecological, evolutionary & environmental sciences

For a reference copy of the document with all sections, see nature.com/documents/nr-reporting-summary-flat.pdf

Life sciences study design

All studies must disclose on these points even when the disclosure is negative.

Sample size	As this study only used published datasets, a sample-size calculation was not conducted. Instead, the authors of the original study determined the sample size.
Data exclusions	No data exclusions were done.
Replication	We successfully replicated our analyses across multiple datasets (N>=2) and presented the results in Figures and Supplementary Figures.
Randomization	As this study only used published datasets, we did not have control over the experimental design, thus, this is not relevant.
Blinding	As this study only used published datasets, we did not have control over the experimental design, thus, this is not relevant.

Reporting for specific materials, systems and methods

We require information from authors about some types of materials, experimental systems and methods used in many studies. Here, indicate whether each material, system or method listed is relevant to your study. If you are not sure if a list item applies to your research, read the appropriate section before selecting a response.

Materials & experimental systems

n/a	Involved in the study	
☒	☐	Antibodies
☒	☐	Eukaryotic cell lines
☒	☐	Palaeontology and archaeology
☒	☐	Animals and other organisms
☒	☐	Clinical data
☒	☐	Dual use research of concern

Methods

n/a	Involved in the study	
☒	☐	ChIP-seq
☒	☐	Flow cytometry
☒	☐	MRI-based neuroimaging
